# Establish a novel tumor budding-related signature to predict prognosis and guide clinical therapy in colorectal cancer

**DOI:** 10.1038/s41598-024-52596-1

**Published:** 2024-01-25

**Authors:** Qixin Li, Gaixia Liu, Quanpeng Qiu, Jiaqi Zhang, Ruizhe Li, Jiamian Zhao, Junjun She, Yinnan Chen

**Affiliations:** 1 Department of General Surgery, The First Affiliated Hospital of Xi’an Jiaotong University, Xi’an, Shaanxi China; 2 Center for Gut Microbiome Research, Med-X Institute, The First Affiliated Hospital of Xi’an Jiao Tong University, Xi’an, Shaanxi China; 3 Department of High Talent, The First Affiliated Hospital of Xi’an Jiaotong University, Xi’an, Shaanxi China

**Keywords:** Colorectal cancer, Tumour biomarkers, Cancer genomics

## Abstract

Tumor budding is a long-established independent adverse prognostic marker for colorectal cancer (CRC), yet assessment of tumor budding was not reproducible. Therefore, development of precise diagnostic approaches to tumor budding is in demand. In this study, we first performed bioinformatic analysis in our single-center CRC patients’ cohort (n = 84) and identified tumor budding-associated hub genes using the weighted gene co-expression network analysis (WGCNA). A machine learning methodology was used to identify hub genes and construct a prognostic signature. Nomogram model was used to identified hub genes score for tumor budding, and the receiver operating characteristic (ROC) curve and calibration plot indicated high accuracy and stability of hub gene score for predicted the prognosis of CRC. The association between budding-associated hub genes and score and prognosis of CRC were further verified in TCGA CRC cohort (n = 342). Then gene set enrichment analysis (GSEA) and gene set variation analysis (GSVA) were applied to explore the signaling pathways related to the tumor budding and validated by immunohistochemistry (IHC) of our clinical samples. Subsequently, immune infiltration analysis demonstrated that there was a high correlation between hub genes score and M2-like macrophages infiltrated in tumor tissue. In addition, somatic mutation and chemotherapeutic response prediction were analyzed based on the risk signature. In summary, we established a tumor budding diagnostic molecular model, which can improve tumor budding assessment and provides a promising novel molecular marker for immunotherapy and prognosis of CRC.

## Introduction

Colorectal cancer (CRC) is among one of the leading causes of cancer-related mortality and morbidity worldwide, responsible for almost 2,000,000 new cases yearly and over 900,000 deaths globally^1^. For decades, cancer staging has relied on anatomy based TNM staging and positive rates of lymph node metastasis ^2^. In the most recent TNM (2017) and WHO (2019) classification schemes, the tumor budding in CRC was emphasized as an additional prognostic factor for this CRC patients^3–5^. Tumor budding, defined as a single cancer cell of up to four cancer cells at the tumor-invasive margin, has emerged as a promising independent prognostic biomarker in CRC^6–8^. Standardization of tumor budding assessment was achieved by the International Tumor Budding Consensus Conference (ITBCC) recommendations in 2016: identification of the budding hotspot (measuring 0.785 mm2) at the invasive front of the tumor and counting of the number of buds results in a score that can be classified as Bd1 (0–4 buds), Bd2 (5–9 buds) or Bd3 (10 or more buds)^9^. The prognostic role of Bd has been widely investigated and currently influences decision making in CRC patients. In patients with pT1 CRC, intermediate to high-grade Bd (Bd2-3) is an independent predictor of lymph node metastasis and is increasingly considered^10,11^. In stage II colorectal cancer, high-grade Bd (Bd3) represents a poor prognostic factor that should warrant consideration of adjuvant chemotherapy^12,13^.

In general, tumor budding has been evaluated by hematoxylin–eosin (H&E) staining, but does not always reproducible^14^. Moreover, positive rates of tumor budding are affected by the selection of slide and pathologists. Therefore, it is of great clinical importance to find additional biomarkers for evaluation and characterization of tumor budding.

Despite the application of machine learning approaches based on deep learning has improved the accuracy of detecting tumor budding, there are still some limitations in those semi-automatic and automatic detection methods^15,16^. The development of a gene signature with an exceptional ability to represent budding levels would help assess the tumor budding in collaboration with pathological sections and overcome poor objectivity in budding judgment.

In the present article, we employed multiple bioinformatic approaches to pick up signature genes of tumor budding in CRC to construct a multigene signature for molecular diagnosis of tumor budding. These genes displayed remarkable diagnostic performance and the prognostic predictive value of the gene signatures was validated in the TCGA cohort. In addition, we investigated the underlying mechanism and immune cell infiltration of tumor budding, and preliminary experimental validation was also performed. Combining traditional methods with molecular markers for evaluation of tumor budding will be helpful for clinical doctors to make correct decision on therapeutic strategies and prevent possible under and overtreatment.

## Results

### Characteristics of patients

**Figure 1:**
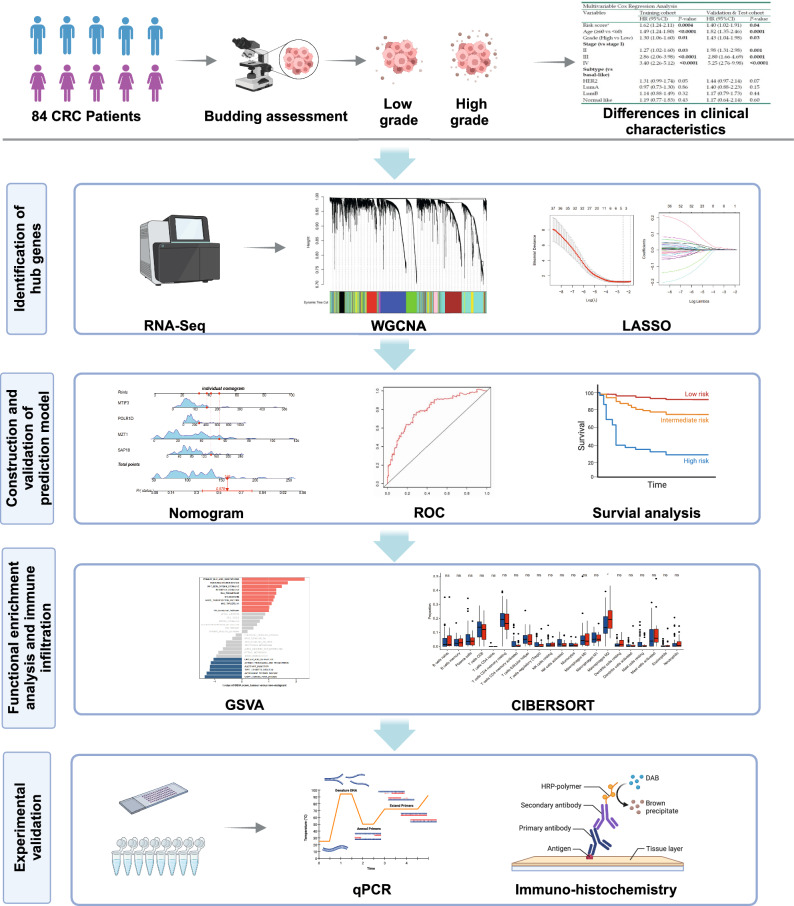
The flow chart of the current study.

**Figure 2:**
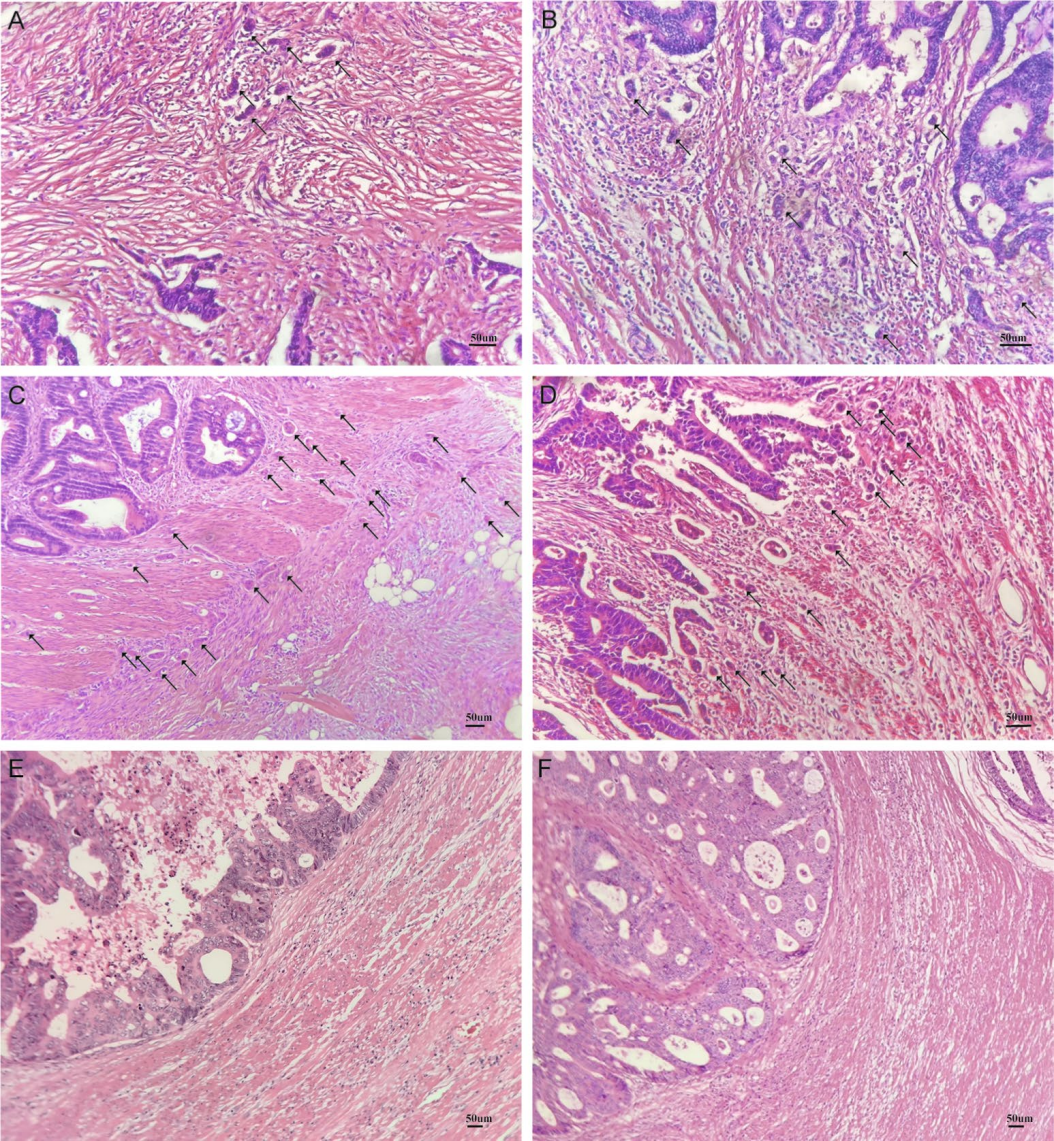
Examples of different tumor budding grades at the invasive front of colorectal cancer (CRC) based on the International Tumor Budding Consensus Conference (ITBCC) 2016. (**A**,**B**): Bd 2. (**C**,**D**): Bd 3. (**E**,**F**):BD 1.

The research flow chart depicts the study design (Fig. 1). After the retrieval of diagnostic glass slides, a total of 84 surgically treated patients diagnosed with CRC were included in the study. Low-grade tumor budding (BD = 1,2) was observed in 70.2% of all cases (n = 59), and high-grade (BD = 3) budding was found in 29.8% (n = 25). Examples of tumor budding assessment in standard H&E staining are presented in Fig. 2. The clinicopathologic characteristics of the 84 patients are summarized in Table1. The significant associations were found between lymph node metastasis (*p* = 0.025), pathological stage (*p* = 0.036) and the grade of tumor budding. In addition, there were no significant differences in distributions of sex, age, smoking status, tumor size and primary site between the two groups.

**Table 1:** The clinicopathological parameters between CRC patients with high- and low-grade tumor budding.

	Tumor budding			
Characteristics	All cases (%)	High-grade (%)	Low-grade (%)	*p*
No. of patients	*84*	25(29.8%)	59 (70.2%)	
Gender				0.882
Female	38	11 (44.0%)	27 (45.8%)	
Male	46	14 (56.0%)	32 (54.2%)	
Age				0.401
Media		64	65	
Range		45–86	48–83	
< 70	62	20 (80.0%)	42 (71.2%)	
≥ 70	22	5 (20.0%)	17 (28.8%)	
Smoking status:				0.327
Never smoker	60	16 (64.0%)	44 (74.6%)	
Smoker	24	9 (36.0%)	15 (25.4%)	
Tumor size (mm)				0.586
< 40 mm	34	9 (36.0%)	25 (42.4%)	
≥ 40 mm	50	16 (64.0%)	34 (57.6%)	
Primary site				0.112
Colon	37	10 (40.0%)	27 (45.8%)	
Rectum	34	8 (32.0%)	26 (44.1%)	
Sigmoid	13	7 (28.0%)	6 (10.2%)	
pT				0.366
pT1 or pT2	13	2 (8.00%)	11 (18.6%)	
pT3 or pT4	71	23 (92.0%)	48 (81.4%)	
pN:				0.025
pN0	46	9 (36.0%)	37 (62.7%)	
pN1 or pN2	38	16 (64.0%)	22 (37.3%)	
Tumor stage diagnoses				0.036
I/II	45	9 (36%)	36 (61.0%)	
III/IV	39	16 (64.0%)	23 (39.0%)	

### Screening for tumor budding-related genes by weighted gene co-expression network analysis (WGCNA) in CRC

**Figure 3:**
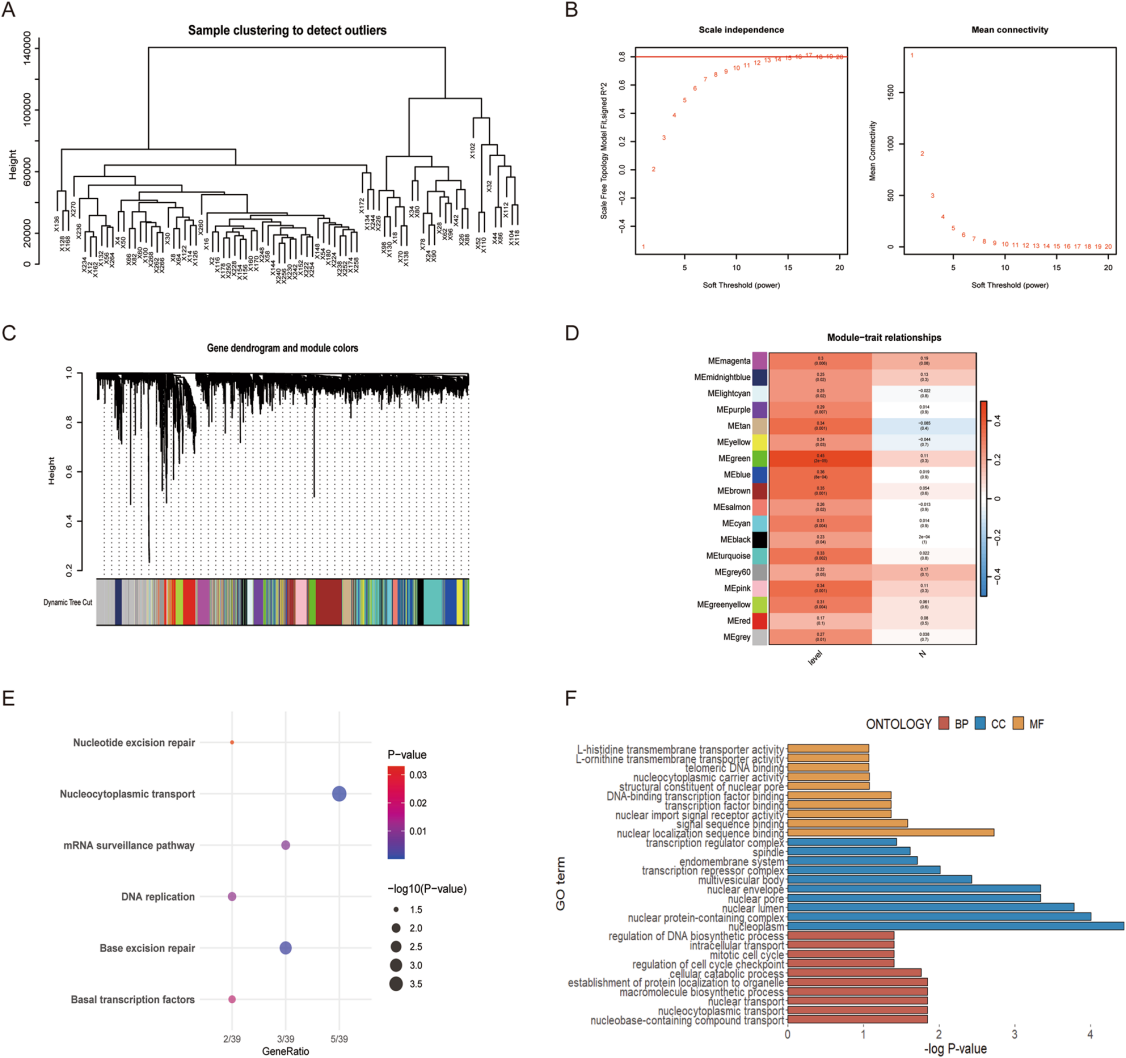
Construction of weighted gene co-expression network. (**A**) Samples were clustered and outlier samples were not found. (**B**) Soft threshold power screening and scale-free network construction. (**C**) Establishment of co-expressed gene modules based on hierarchical clustering algorithm. (**D**) Correlation of gene modules with tumor budding and pN stage. (**E**,**F**) KEGG^31–33^ and GO enrichment analysis of the green module.

Correlation networks are used for identifying clusters of highly correlated genes across colorecta tumor tissues. WGCNA was performed using the expression profiles of the top 50% of variance in the 84 CRC patients cohort, and no outlier was detected in the cohort (Fig. 3A). The adjacency matrix was constructed based on the criterion of gene distribution that conformed to a scale-free network when setting the soft-threshold power of β = 16 (R2 = 0.80), retaining high connectivity information (Fig. 3B). The dynamic cut tree was made after merging similar gene modules, with the number of genes per module not less than 50 (Fig. 3C, D). Among the 18 gene module, the green module is associated most significantly with tumor budding level (correlation = 0.45, *p* < 0.001). Furthermore, we performed a Gene Ontology (GO) enrichment and Kyoto Encyclopedia of Genes and Genomes (KEGG) signaling pathway analysis for the green module. The GO enrichment results indicated that the genes in green model were primarily related to telomeric DNA binding and regulation of cell cycle checkpoint, among others (Fig. 3E). The analysis of KEGG pathway suggested that the genes were involved in the Nucleocytoplasmic transport and Base excision repair (Fig. 3F).

### Construction and validation of a 4-gene risk signature and nomogram for tumor budding

**Figure 4:**
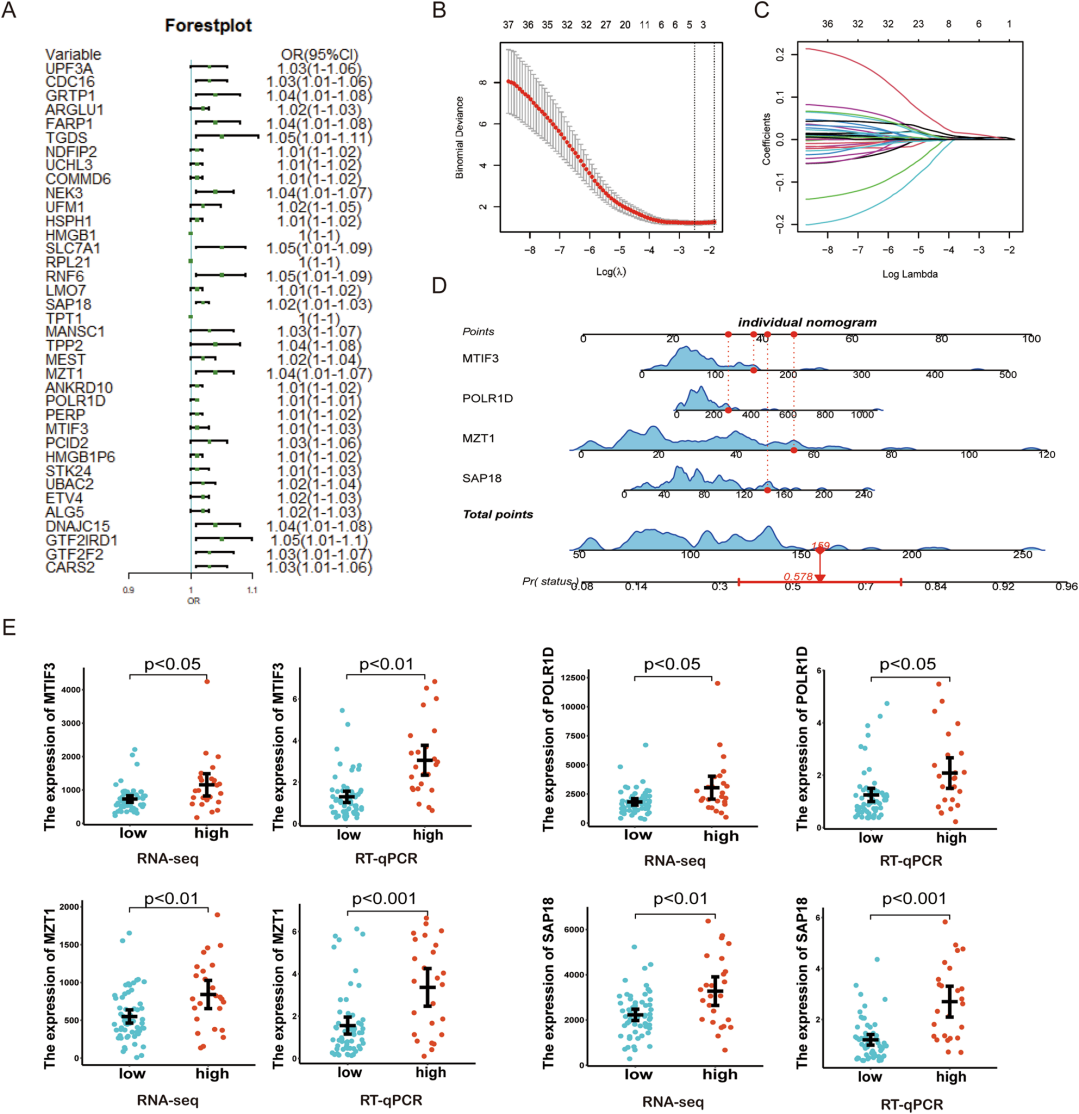
The development of a nomogram for diagnosing tumor budding in CRC. (**A**) Univariate analyses for predicting tumor budding in the CRC cohort. (**B**,**C**) Four genes were selected to construct the diagnosis model by least absolute shrinkage and selection operator (LASSO) regression. (**D**) Comparison of expression of 4-genes between low- and high-grade tumor budding group.

To build an efficient model to identify tumor budding in CRC,38 genes were determined by univariate logistic regression with *p* < 0.01 filtering from genes in green module (Fig. 4A). The least absolute shrinkage and selection operator (LASSO) regression analysis was employed to reduce the number of candidate genes using the lowest value of lambda λ to avoid the risk of over-fitting (Fig. 4B,C), and 4 candidate genes with the most powerful predictive features were identified (MTIF3,POLR1D,MZT1 and SAP18). We then constructed a nomogram and scored risk of tumor budding based on the 4 candidate genes (Fig. 4D). We also observed that the mRNA expression levels of the four genes in tumor budding high-grade group generally exhibited higher compared to low-grade group (Fig. 4E). Meanwhile, we performed qPCR to validated the results from RNA-seq (low-grade budding n = 59, high-grade budding n = 25) (Fig. 4E).

**Figure 5:**
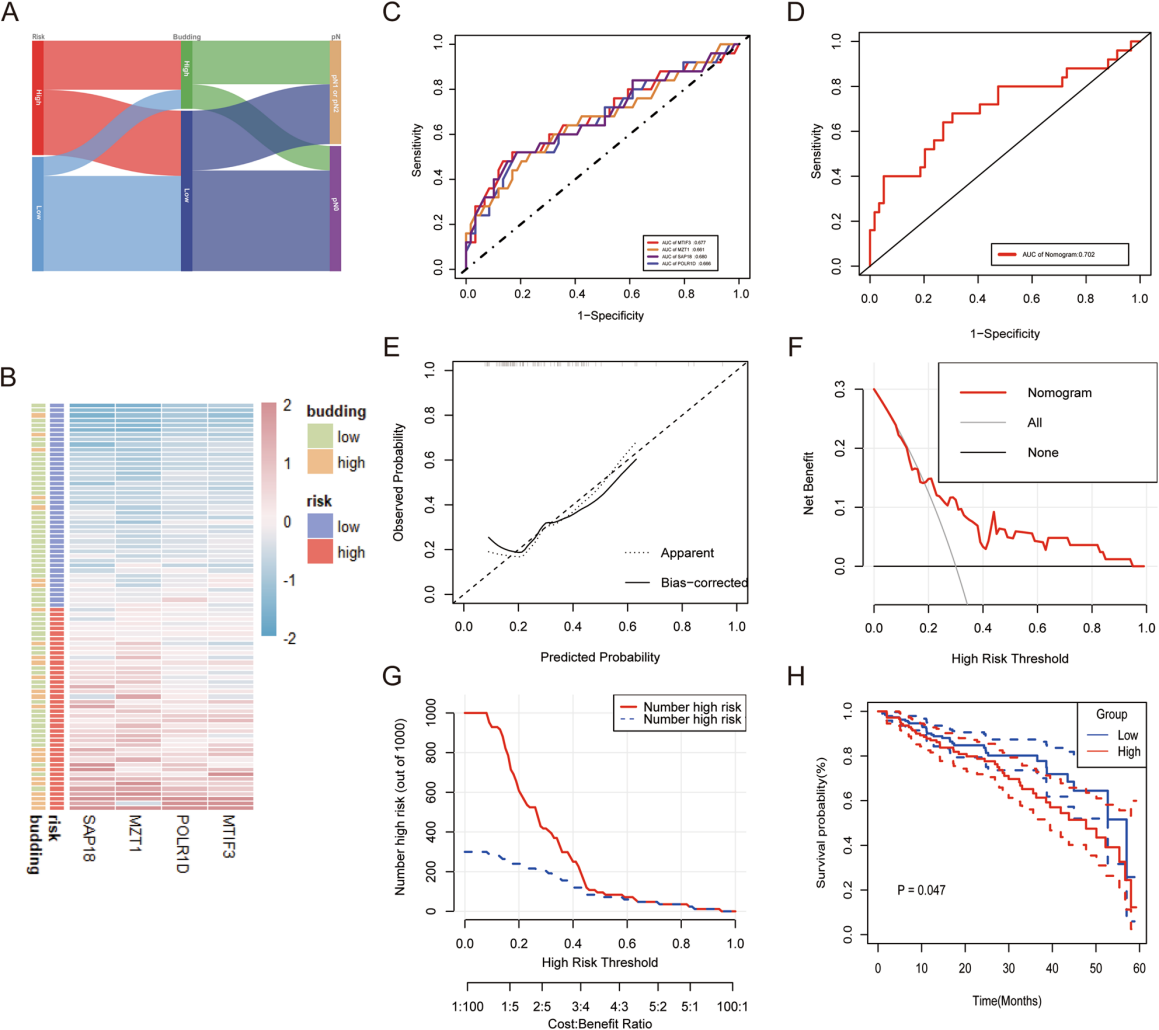
Validation of the nomogram. (**A**) The ggalluvial diagram displaying relationships among tumor budding grade, risk score and pN. (**B**) Expression patterns of 4 hub genes in high- and low-risk groups. (**C**,**D**) receiver operating characteristic (ROC) analysis of the 4 hub genes and nomogram for tumor budding diagnosis. (**E**) Calibration curve for nomogram. (**F**) The decision curve analysis (DCA) curves of the nomograms. (**G**) Decision curve for nomogram. (**H**) Kaplan–Meier analysis of the overall survival (OS) between the high- and low-risk CRC patients in TCGA cohort.

In the Sankey diagram, we identified the interrelationship between the risk score ,tumor budding and pN stage, and the patients with high risk score were mainly concentrated in high grade tumor budding (Fig. 5A). The heatmap visualized the detailed expression of budding-related genes in the high and low risk score group (Fig. 5B). To further assess the predictive efficiency of the diagnostic column line graph model, the receiver operating characteristic (ROC) curve was established to assess the diagnostic specificity and sensitivity of each gene and the nomogram. We calculated the area under the curve (AUC) for each item. The results were as follows: MTIF3 (AUC 0.677), MZT1 (AUC 0.661), SAP18 (AUC 0.680), POLR1D (AUC 0.666), and nomogram (AUC 0.702) (Fig. 5C, D). These findings imply that all major genes are involved in tumor budding, and the constructed nomogram had the highest diagnostic value. According to the calibration curve, the error between the actual risk and the predicted risk was very small (Fig. 5E). The decision curve analysis (DCA) revealed that the nomogram had a higher accuracy that can provide evidence for clinical decisions (Fig. 5F). Based on the results of DCA, we further plotted clinical impact curves to evaluate the clinical utility of the nomogram. The ‘Number high risk’ curve was close to the ‘Number high risk with event’ curve at the risk threshold from 0.4 to 1, which demonstrated that the nomogram owned powerful predictive ability (Fig. 5G). We compared risk scores between high-grade and low-grade tumor budding groups in the 84-CRC cohort. The results showed that the risk score of the high-grade budding group was significantly higher than that of the low-grade group (Supplementary Figure S1). Subsequently, we evaluated the correlation between gene signature of tumor budding (risk score) and prognosis of the colon adenocarcinoma (COAD) and rectum adenocarcinoma (READ) patients from the TCGA. We classified CRC patients into high (n = 143) and low-risk (n = 199) subpopulations with the mean value of risk score. Survival analysis uncovered that low-risk CRC individuals displayed a remarkable survival advantage (Fig. 5H). Additionally, we conducted a comparison of clinical characteristics between the high- and low-risk tumor budding groups in the TCGA cohort and observed significant differences in lymph node metastasis (*p* = 0.001) and pathological stage (*p* = 0.002), consistent with our findings from our 84-CRC cohort (Supplementary Table S1).

### Wnt beta catenin signaling and hedgehog signaling are highly correlation with tumor budding

**Figure 6:**
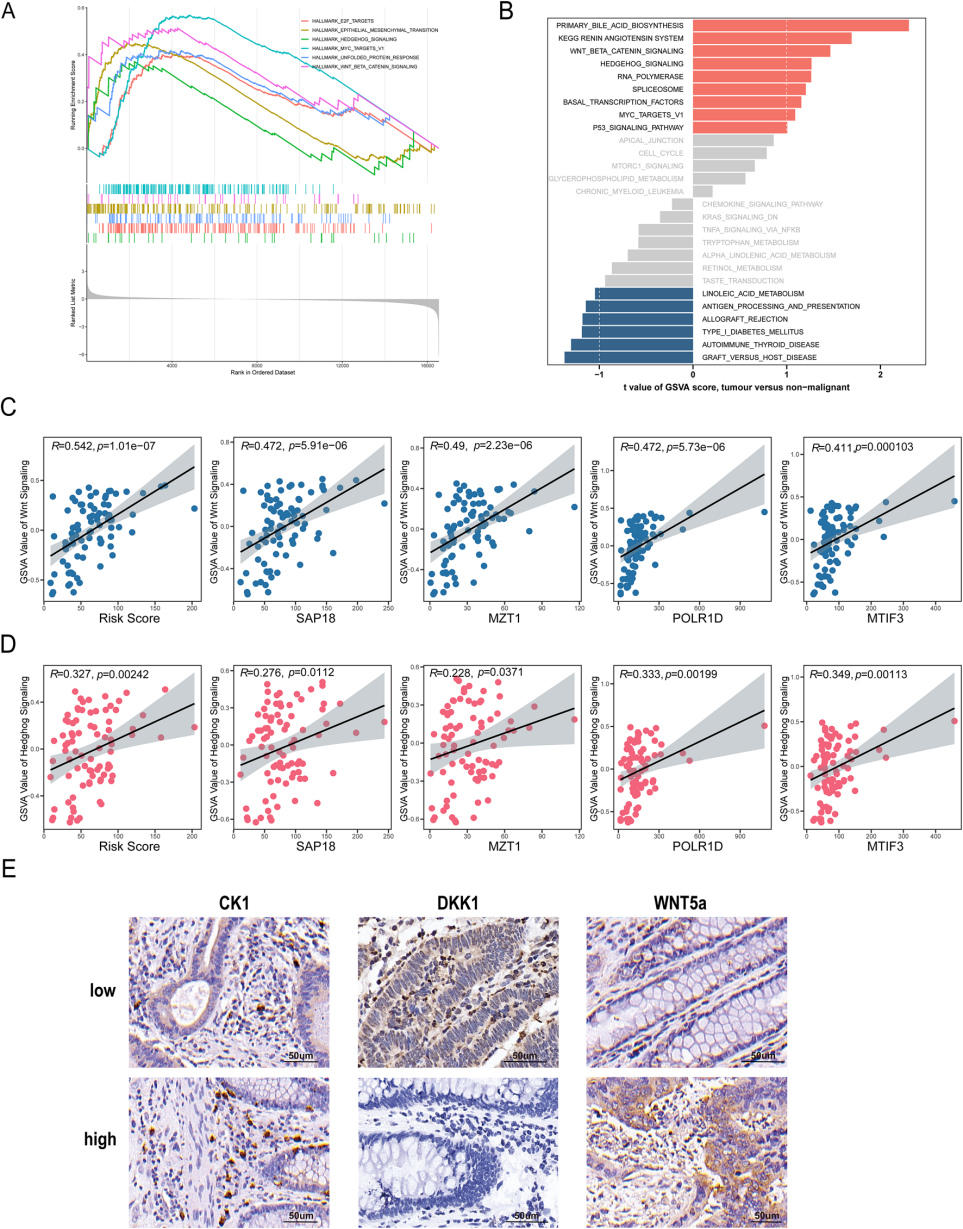
Signaling pathways associated with tumor budding. (**A**) Enrichment pathways between low-and high-grade tumor budding groups through GSEA enrichment analysis. (**B**) The differential enrichment of biological processes and pathways between low-grade and high-grade tumor sprouting groups was demonstrated through GSVA analysis, using KEGG^31–33^ and HALLMARK databases. Correlation between risk score and enrichment scores of wnt beta catenin signaling (**C**) and hedgehog signaling (**D**). (**E**) Immunohistochemical data of CK1, DKK1 and WNT 5a in low-and high-grade tumor budding groups.

To further elucidate the possible mechanisms underlying tumor budding, we performed gene set enrichment analysis (GSEA) and gene set variation analysis (GSVA) using HALLMARK gene sets and KEGG signaling pathways in our 84-CRC cohort (low-grade budding n = 59, high-grade budding n = 25). The GSEA enrichment analysis revealed six pathways with significant enrichment (*p* < 0.05), including E2F targets, epithelial mesenchymal transition, hedgehog signaling, MYC targets V1, unfolded protein response, and Wnt beta-catenin signaling, as depicted in Fig. 6A. Furthermore, we performed GSVA to analyze differentially enriched pathways between low- and high-grade budding groups. The results revealed a significant enrichment of the primary bile acid biosynthesis, renin angiotensin system, wnt beta catenin signaling, as well as hedgehog signaling and RNA polymerase (Fig. 6B). We discovered that wnt beta catenin and hedgehog signaling enriched in high-grade tumor budding were detected in GSEA and GSVA. Subsequently, we verified that the risk score and 4-gene was related to the enrichment scores (ESs) of the GSVA in wnt beta catenin signaling and hedgehog signaling. As shown in Fig. 6C, D, the risk score was positively correlated with wnt beta catenin and hedgehog signaling. Several studies have reported that epithelial mesenchymal transition (EMT) plays a pivotal role in tumor budding. Therefore, the EMT scores calculated by gene set variation analysis (GSVA) were compared between the high-grade and low-grade tumor budding groups (Supplementary Figure S2). The results demonstrated a significantly higher EMT score in the high-grade tumor budding group compared to the low-grade group (*p* < 0.05). To further validate the association between tumor budding and wnt beta catenin signaling, we analyzed the expression of positive regulators (CK1 and wnt5a) and the negative regulator (DKK1) of wnt beta catenin signaling in CRC tissues (n = 84) by immunohistochemistry (IHC) staining (Fig. 6E, Supplementary Figure S3 and Table S2).The results revealed significant elevation of CK1 and wnt5a in high-grade tumor budding tissues compared with low-grade tumor budding tissues, while DKK1 was highly expressed in low tumor budding group. Overall, these results indicated that high risk-scores of the tumor budding were associated with activation of the wnt beta catenin and hedgehog signaling pathways.

### M2-like macrophages cells were enriched in CRC tissue with high-grade tumor budding

**Figure 7:**
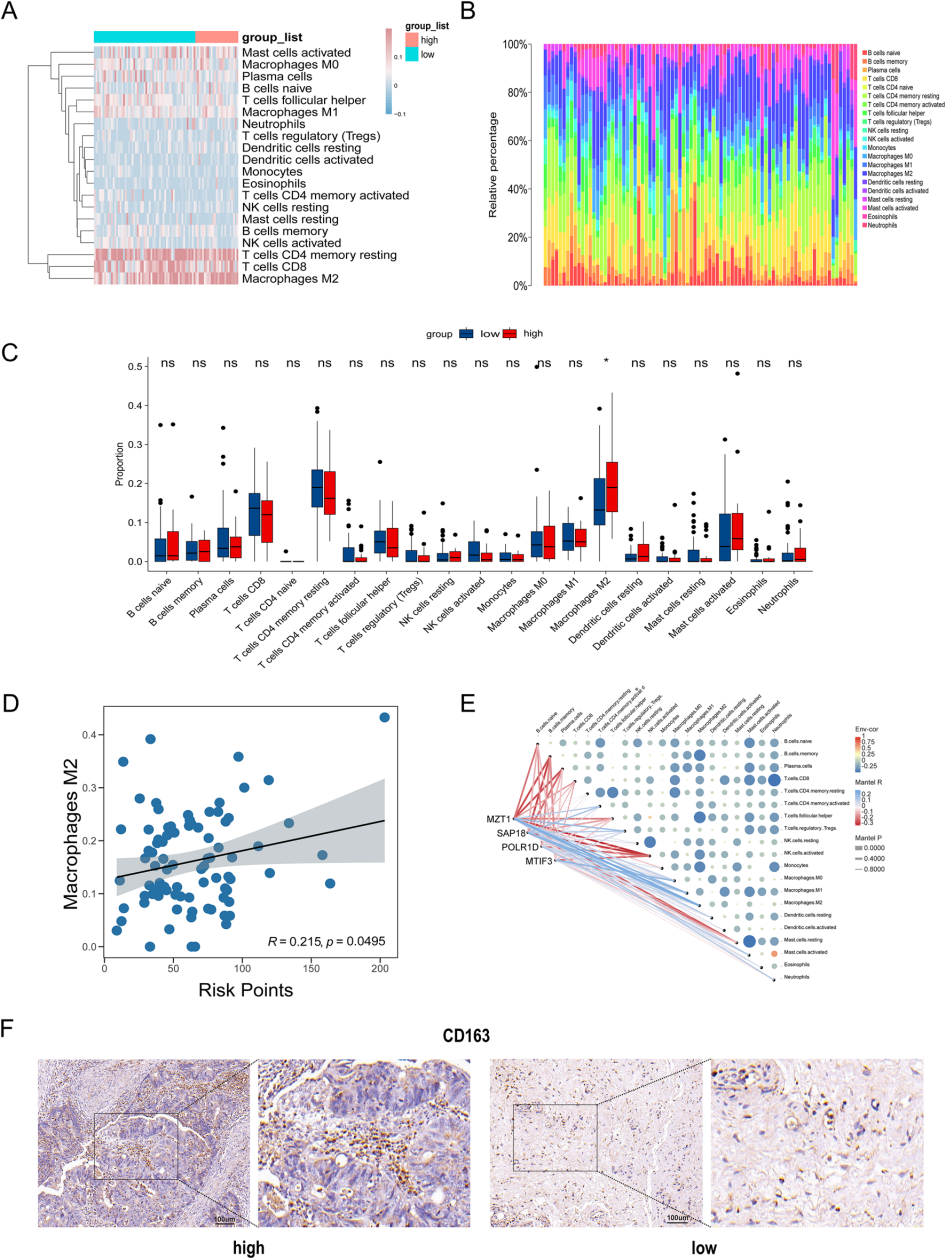
Immune cell infiltration and correlation analysis of Hub genes with immune cells. (**A**) Stacked plot of the expression of 22 types of immune cells in each sample. (**B**,**C**) Differences in immune cell levels between different tumor budding grade groups. (**D**) Correlation between risk score and M2-like macrophages cells in CRC tissues. (**E**) Correlation between immune cells and 4-hub genes. (**F**) Immunohistochemical data of CD163 in low-and high-grade tumor budding groups.

The relationship between the tumor immunological microenvironment (TIM) and tumor development is well-established. To explore the landscape of the immunological microenvironment in patients with high and low grade of tumor budding in CRC, we used the CIBERSORT R script to assess the relative proportions of diverse immune cell populations within each CRC sample (Fig. 7A). Notably, a pronounced disparity between the high-grade and low-grade groups in the infiltration of M2-like macrophages cells was identified (Fig. 7B, C). Simultaneously, we stratified the cohort into high and low groups based on the calculated M2-like macrophages infiltration fraction and performed a chi-square analysis with respect to tumor budding grade (Supplementary Table S3). The results revealed a persistent significant difference (*p* = 0.05). Subsequently, we found a positive correlation between the proportion of M2-like macrophages cells and the risk score (R = 0.22, *p* < 0.05) (Fig. 7D). To further understand the role of 4-genes signature in immune infiltration, we used Spearman correlation analysis to determine whether they are associated with immune cell infiltration (Fig. 7E). We observed that genes SAP18 and MZT1 were positively correlated with M2-like macrophages, while all of 4-genes negatively correlated with memory B cells and activated NK cells. To further validate the association between tumor budding and infiltration of M2-like macrophages, we analyzed the densities of M2-like macrophages in CRC tissues by IHC staining (Fig. 7F, Supplementary Figure S3 and Table S2). IHC images showed that colorectal carcinoma tissue from patient with low-grade tumor budding possess lower density of M2-like macrophages compared with CRC tissue from high-tumor budding group. Collectively, these results indicated that tumor budding was associated with infiltration of M2-like macrophages in CRC.

### CRC tissue with high-grade tumor budding was characterized by higher frequencies of APC and TP53 mutations and drug resistance

**Figure 8:**
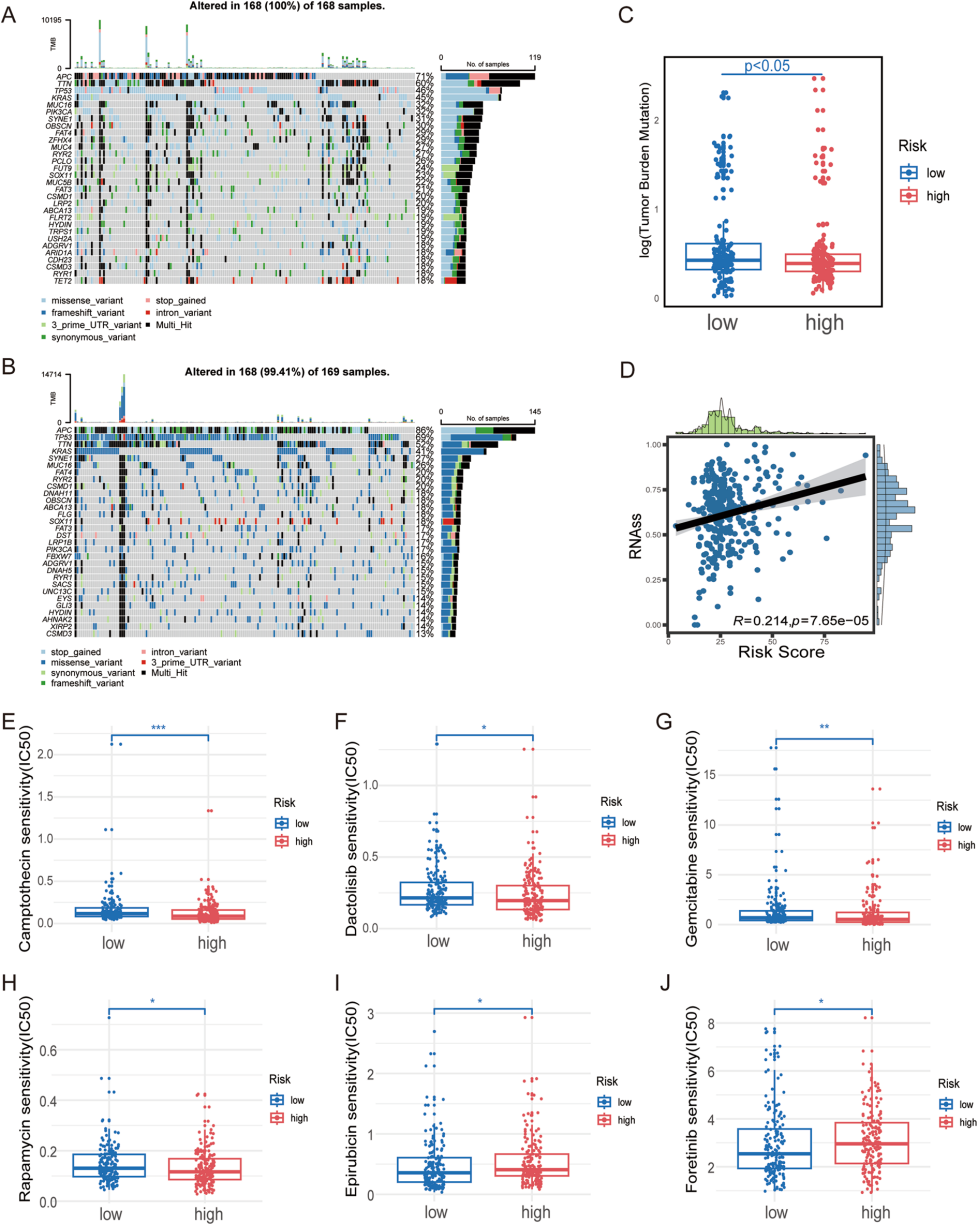
4-gene signature for tumor budding are related to Somatic mutation and drug sensitiveness. (**A**,**B**) The waterfall plot of somatic mutation characteristics in high- and low-risk score groups. (**C**) Differential analysis of tumor mutation burden (TMB) in distinct risk score groups. (**D**) Relationships between risk score and cancer stem cell (CSC) index. (**E**–**J**) Relationships between risk score and chemotherapeutic sensitivity. *p < 0.05; **p < 0.01; ***p < 0.001; ****p < 0.0001; ns, no statistical significance.

To identify the difference in cancer-related gene mutations between the high-risk and low-risk groups, we analyzed the distribution variations of the somatic mutations between two risk score groups in the TCGA COAD/READ cohort (Fig. 8A, B). Comparison of the mutant genes in the two groups revealed that patients with a high-risk score had markedly higher frequencies of APC and TP53 mutations. Our analysis of the mutation data from the TCGA COAD/READ cohort demonstrated that a lower tumor mutation burden (TMB) was observed in the sets of high risk score than that in the sets of low risk score (Fig. 8C). Accumulative evidence shows that patients with a high TMB may benefit from immune checkpoint inhibitors (ICIs)^17^. In addition, cancer stem cells (CSCs) have been recognized as promising therapeutic targets for cancer therapy^18^. As a result, we first assess the potential correlation between the 4-gene risk score and the cancer stem cell (CSC) in TCGA COAD/READ cohort. Figure 8D showed the results of the positive linear correlation between risk score and CSC index (R = 0.214, *p* < 0.001), indicating that CRC cells with higher risk score had more distinct stem cell properties and a lower degree of cell differentiation. We next use drug information from the GDSC database to calculate the half-maximal inhibitory concentration (IC50) values of 198 chemotherapy drugs or inhibitors. We found that the patients in the high-risk score group had lower IC50 value for Camptothecin, Dactolisib, Gemcitabine and Rapamycin, while IC50 values of chemotherapeutics such as Epirubicin and Foretinib were significantly lower in the patients with a low-risk score (Fig. 8E–J).

## Discussion

Accumulating evidence has demonstrated that tumor budding has been associated with tumors with aggressive biology, and has been independently associated with poor outcome, lymph node metastasis, and high recurrence^19^. Identifying reliable and effective biomarkers for tumor budding is indispensable. However, studies on gene signature of tumor budding in colon cancer remain limited.

In this study, we used the WGCNA method to identify tumor budding-related genes. Simultaneously, LASSO-Cox was used to filter genes to construct the 4-gene signature containing: MTIF3, POLR1D, MZT1 and SAP18. The RNA-seq data utilized in this study were exclusively derived from tumor bulk tissues rather than tumor budding cells. Our hypothesis posits that tumor budding represents not merely a cellular variation, but rather an indication of the entire tumor tissue being predisposed to bud and subsequently metastasize. Obtaining transcriptome data from tumor bud cells is considerably more challenging and costly in clinical practice compared to acquiring such data from tumor bulk tissues. Therefore, the 4-gene signature may possess enhanced clinical significance in augmenting the diagnostic efficacy of tumor budding. However, the relation between the 4-gene signature and tumor budding in CRC remains unknown. RNA polymerase I subunit D (POLR1D), encoding a subunit of both RNA polymerase I and III, has been shown to be overexpressed in several human cancer types. It has been reported that POLR1D was positively correlated to tumor size and poor survival of CRC patients^20^. Aberrant expression of POLR1D significantly promoted cell proliferation and migration in vitro, and influenced tumor growth in vivo. In our study, we also found that POLR1D was highly expressed in the high tumor budding group. These suggested that POLR1D may function as a risk factor for predicting the outcome of CRC patients, but the relationship between POLR1D and tumor budding needs to be further explored. Furthermore, to our knowledge, the role of the remaining gene signature in tumor budding has not been reported in the literature. Given that our primary research objective is to screen biomarkers capable of predicting tumor sprouting, this study did not delve into this issue extensively. In order to enhance the comprehension of the underlying mechanisms, a more comprehensive investigation will be conducted in subsequent studies.

We then established and verified a tumor budding-related diagnostic model that can accurately anticipate the risk of tumor budding in two CRC cohorts. Furthermore, four hub genes were utilized to construct a nomogram model and scored risk of tumor budding. The analysis of diagnostic value indicated that our diagnostic model possessed high accuracy and stability for tumor budding diagnosis, which implied great potential for clinical translation. Notably, the constructed nomogram model showed higher accuracy than four candidate biomarker genes alone. In summary, the combination of our genetic diagnostic models and H&E staining sections may reduce the low miss rate and improve the precision in tumor budding assessment, which will contribute to the development of more personalized and precise individualized therapy. Next, we explore the molecular characteristic of CRC with high grade tumor budding.

For signaling pathway related to tumor budding, we used GSEA and GSVA to explore the signaling pathways affecting the tumor budding. We found that wnt β-catenin and hedgehog signaling were simultaneously enriched in both analyses. Tumor budding has long been hypothesized to be comprised of cells undergoing the epithelial–mesenchymal transition (EMT), while the wnt pathway is considered to be the key modulator of EMT^21^. However, more experiments required to explore the relationship between wnt β-catenin signaling and tumor budding in CRC.

For immunological microenvironment, we carried out a differentiation of the contents of immune cells in high and low-grade tumor budding groups utilizing the CIBERSORT. The findings determined that the high-grade group had substantially elevated infiltrative levels of M2-like macrophages. Investigations have shown that in most tumor microenvironments, M2-like macrophages are engaged in inflammation resolution and suppress tumor cell immunity, thereby promoting cancer progression and metastasis^22,23^. This suggests that M2-like macrophages may play a facilitative role during the development of tumor budding.

Furthermore, our study showed that CRC tissue with high-grade tumor budding was characterized by higher frequencies of APC and TP53 mutations and drug resistance. This finding, although not unexpected, substantiates the robustness of our gene model and contributes valuable insights for further investigation into the underlying mechanisms governing tumor sprouting.

In conclusion, we first established a tumor budding diagnostic molecular model based on our CRC cohort, 4-gene risk score was conducted to evaluation and characterization for tumor budding. The diagnostic model possessed high accuracy and stability for tumor budding diagnosis was validated in our CRC cohort and TCGA CRC cohort. We also explore the signaling pathway (wnt β-catenin signaling, hedgehog signaling) and immune cells (M2-like macrophages) enriched in CRC with high-grade tumor budding, which indicate the underlying mechanism of tumor budding. Our study improves tumor budding molecular assessment and provides a promising novel molecular marker for immunotherapy and prognosis of CRC.

## Materials and methods

### Study design

We first conducted a CRC patients’ cohort from The First Affiliated Hospital of Xi’an Jiao tong University, which enrolled all consecutive CRC patients with histologically confirmed adenocarcinoma from October 2022 to June 2023. Of these, 97 patients had diagnostic glass slides available for analysis and were included in the study. Our exclusion criteria included: 1. CRC Patients with age ≤ 18 years old (n = 4); 2. CRC Patients with hereditary syndromes (e.g., Lynch syndrome and familial adenomatous polyposis) (n = 4); 3. Patients with any malignant disease history, inflammatory bowel disease (IBD) history before the diagnosis of colorectal cancer (n = 2); 4. Patients with multiple primary colorectal carcinoma (n = 3). Finally, 84 CRC patients were included in this study for further analysis. The study was approved by the institutional review board of The First Affiliated Hospital of Xi'an Jiao Tong University. All patients signed an Institutional Review Board-approved written informed consent.

### Data collection

The clinical data of all included patients were obtained from Biobank of the First Affiliated Hospital of Xi’an Jiao Tong University. Paraffin-embedded tissue blocks of all 84 patients were retrieved from the archives of the Department of Pathology, The First Affiliated Hospital of Xi'an Jiao Tong University. All histomorphological data were reviewed from the corresponding H&E-stained slides. This study was conducted in accordance with the Declaration of Helsinki and was approved by the institutional review board of The First Affiliated Hospital of Xi'an Jiao Tong University. All clinical data involved in the present study was anonymized and de-identified prior to analysis. Informed consent was obtained from all subjects involved in the study.

### Definition and evaluation of tumor budding

Tumor cell clusters of four or less tumor cells infiltrating the adjacent parenchyma were defined as budding as previously described. The grade of budding was reviewed with the same microscope by two independent pathologists blinded to the clinical and outcome data through hematoxylin–eosin (H&E) staining. Evaluation of tumor buds was performed as described by the ITBCC 2016. In case of a discrepancy between the two pathologists, the overall result was decided by consensus. Patients were subsequently grouped into two budding grades as Low Grade (budding categories 1) and High Grade (budding category 2 and 3) for subsequent analyses.

### RNA sequencing

Total RNA was extracted from human colorecta tumor bulk tissues. The generation and sequencing of cDNA libraries were performed on the Illumina sequencing platform (Nova) to generate 150 bp paired-end reads. Clean RNA-seq reads were mapped to human reference genome (hg38) along with the gene annotation data (genecode v29) from the Genecode database using STAR (v2.5.3a). For the TCGA cohort, clinical features, RNA-seq expression data [fragments per kilobase million (FPKM) value] were downloaded from the TCGA database (https://cancergenome.nih.gov/).

### Weighted gene coexpression network analysis

We used WGCNA which can convert coexpression correlation into connection weights or topological overlap values, to identify coexpressed genes in tumor budding. Network type was set as the “unsigned” type. We used standard deviation (SD > 50%) to screen highly variable genes in the WGCNA expression data. A total of 84 samples was used as an expression matrix for further analysis. The soft thresholding power was chosen as 5 to construct a gene network and calculate coexpression similarity and adjacency, which was transformed into a topological overlap matrix (TOM). Hierarchical clustering based on TOM was used to cluster the modules. We used Pearson’s test to calculate the correlation between module eigengenes (MEs) and tumor budding. When *p* < 0.05, the module was considered to be significantly related to tumor budding^24^.

### Identification of distinct genes

We identified candidate hub genes by the key module genes. Then, univariate logistic regression and LASSO–Cox regression analysis was applied to these prognostic candidates. Lasso regression is a method based on linear regression that can be used to select the features that are most relevant to the target variable. It compresses the value of certain coefficients by punishing the L1 norm, thereby reducing unnecessary features in the model. Finally, by choosing the optimal penalty parameter λ correlated with the minimum tenfold cross-validation, we established a four-gene optimal prognostic model. Subsequently, the rms algorithm was used to construct a nomogram model that predicted the probability of tumor budding.

### Diagnostic column line graph construction and validation

We created a column line graph model to predict the tumor budding using the “rms” program. In this process, we can obtain a tumor budding risk score based on four hub genes for each sample, and then we verified the diagnostic value of this tumor budding risk score for tumor budding in the ROC curve and calibration curves. Finally, decision curve analysis and clinical impact curves were used to assess the clinical utility of the model. The prognostic risk model was validated using TCGA-COAD-READ cohorts^25^.

### Functional enrichment analysis

Biological functional enrichment was analyzed using GO analysis and the KEGG pathway based on The Database for Annotation, Visualization and Integrated Discovery (DAVID) database (https://david.ncifcrf.gov/ (accessed on 29 September 2022)). The cutoff criterion was defined as *p* < 0.05^26^. KEGG and Hallmaker pathways were analyzed using the R packages "GSVA" and "msigdbr" to explore biological process differences between high-grade and low-grade tumor budding groups in our 84-CRC cohort.

### Tumor immune analysis

The proportion of 22 different types of immune cells in our CRC cohort (n = 84) was calculated through Cibersort. A Spearman-related analysis of infiltrating immune cells with Hub genes was calculated using “Corrplot” in R. *P* < 0.05 was considered statistically significant.

### Mutation and drug susceptibility analysis

To identify somatic mutations in CRC patients belonging to high- and low-risk groups, the 'maftools' R package was utilized for the analysis of whole genome re-sequencing data from the CRC cohort (n = 337) in the TCGA database. Additionally, we computed the tumor mutational burden (TMB) score for each CRC patient in both risk groups. Furthermore, we investigated the association between these risk groups and cancer stem cells (CSCs). In order to assess potential differences in therapeutic efficacy of commonly used chemotherapeutic drugs for CRC patients in these two risk groups, we determined the IC50 values using the "oncoPredict" package^28^.

### Real-time polymerase chain reaction (qRT–PCR)

Total RNA was extracted using TRIzol reagent (Invitrogen, Thermo Fisher Scientific, Inc.) from tumor tissue. The PrimeScript™ RT reagent kit (TaKaRa) was used for reverse transcriptase reaction. RNA levels were determined by quantitative real-time PCR (qRT-PCR) in triplicate on a Bio-Rad CFX96 using the SYBR Green method (RR420A, Takara, Mountain View, CA, USA).The primers of the five genes were listed in Table S3. Fold differences were calculated for each group using normalized CT values^29^. The primer sequences are listed in Supplementary Table S4.

### Immunohistochemical analysis

Through IHC experiments, we detected the protein expression of CK1, DKK1, WNT 5a and CD163 in paraffin sections of human colon tissues. A total of 84 CRC tissues were obtained from The First Affiliated Hospital of Xi’an Jiao tong University. All the patients signed an informed consent form. The IHC staining results were interpreted by both the intensity of staining and the staining positive area, and were independently reviewed by two independent pathologists. The percentage of positive cells was scored as follows: < 5%, 0 point; 5–25%, 1 point; 26–50%, 2 points; 51–75%, 3 points; 76–100%, 4 points. The staining intensity evaluation criteria were as follows: 0 point for colorless; 1 point for pale yellow; 2 points for tan; 3 points for brown. The final IHC staining score for each tissue was obtained by multiplying the scores of positively stained cells and the scores of staining intensities.

### Statistical analysis

R (version 4.0.5) was applied to perform statistical analysis in our study. χ2 test was used to obtain *p*-values when comparing categorical variables. Continuous variables were compared, and *p*-values were obtained by T test. The correlation matrices were conducted using Pearson or Spearman correlation. Wilcoxon test was conducted for the comparisons between the two groups. Survival differences were compared using K–M curves with a Log-rank test. All statistical tests were two-sided, and *p* < 0.05 was considered as statistical difference^30^.

### Supplementary Information

Supplementary Figure S1.

Supplementary Figure S2.

Supplementary Figure S3.

Supplementary Table S1.

Supplementary Table S2.

Supplementary Table S3.

Supplementary Table S4.

## Data Availability

The raw sequence data reported in this paper have been deposited in the Genome Sequence Archive (Genomics, Proteomics & Bioinformatics 2021) in National Genomics Data Center (Nucleic Acids Res 2022), China National Center for Bioinformation/Beijing Institute of Genomics, Chinese Academy of Sciences (GSA-Human: HRA005817) that are publicly accessible at https://ngdc.cncb.ac.cn/gsa-human.
