# Supplementary figures and images for: Establish a novel tumor budding-related signature to predict prognosis and guide clinical therapy in colorectal cancer
Source: Sci Rep. 2024 Jan 25;14:2180. doi: 10.1038/s41598-024-52596-1 (PMC10810877; doi:10.1038/s41598-024-52596-1)

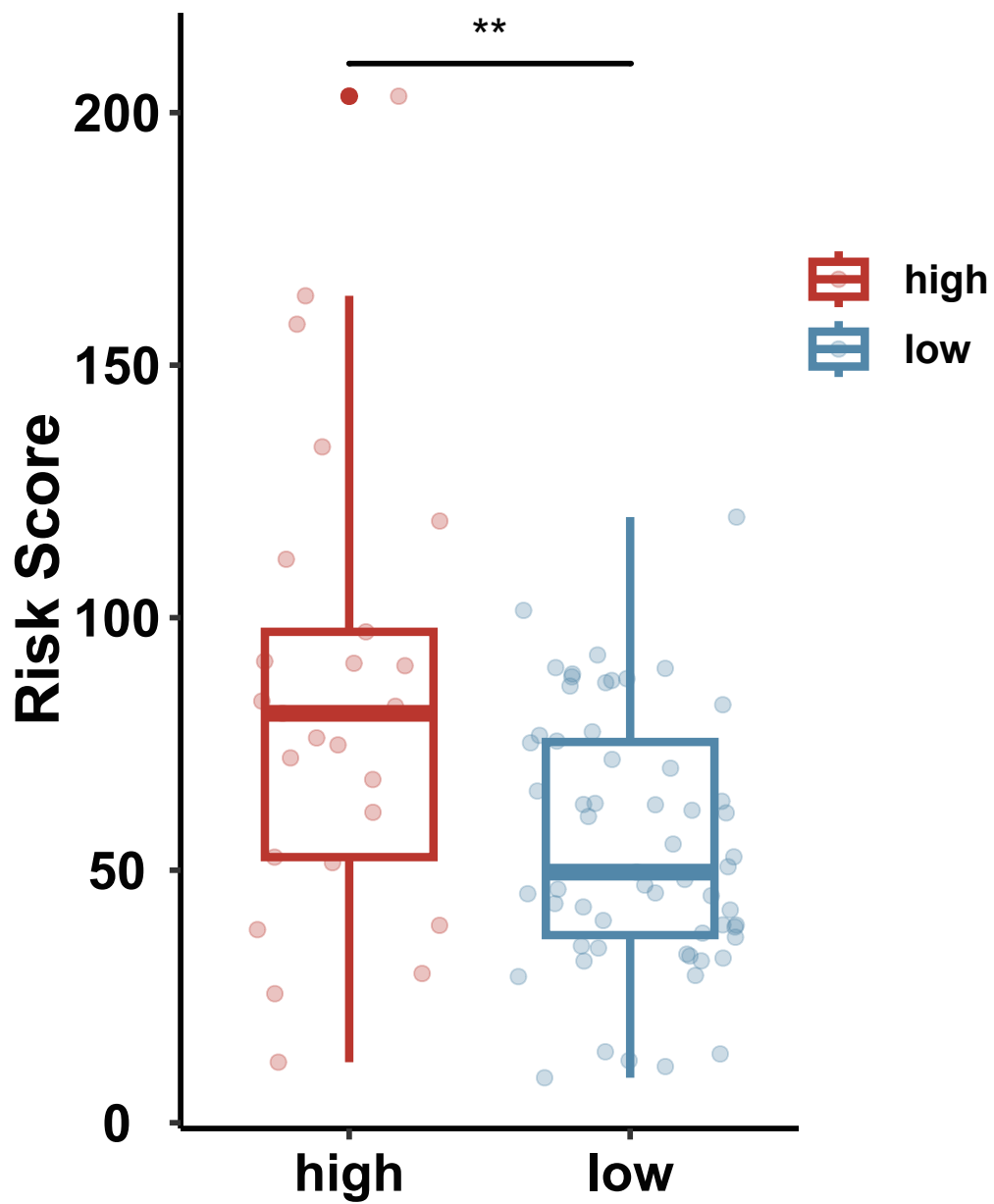

Figure S1. Risk scores for budding of high-grade and low-grade tumors

Supplement: Supplementary file 1 — Supplementary Figure S1. [file 41598_2024_52596_MOESM1_ESM.pdf]

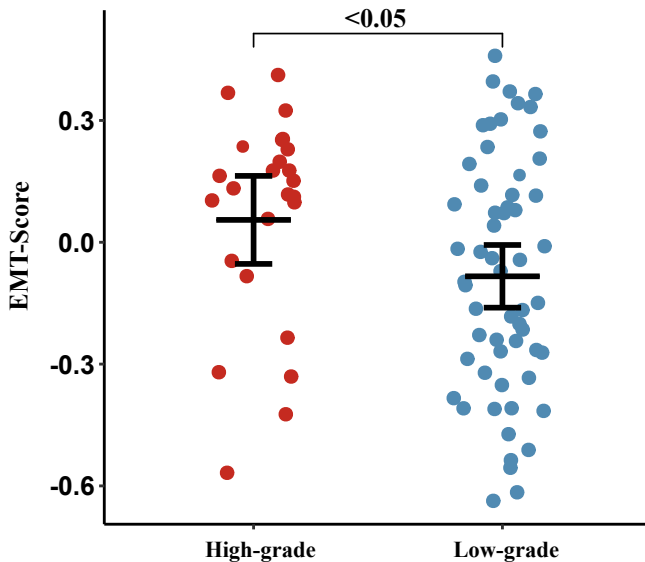

FigureS2.Comparison of the EMT score between high- and low-grade tumor budding groups.

Supplement: Supplementary file 2 — Supplementary Figure S2. [file 41598_2024_52596_MOESM2_ESM.pdf]
