# Supplementary material for: Establish a novel tumor budding-related signature to predict prognosis and guide clinical therapy in colorectal cancer
Source: Sci Rep. 2024 Jan 25;14:2180. doi: 10.1038/s41598-024-52596-1 (PMC10810877; doi:10.1038/s41598-024-52596-1)
Supplement: Supplementary file 3 — Supplementary Figure S3. [file 41598_2024_52596_MOESM3_ESM.pdf]

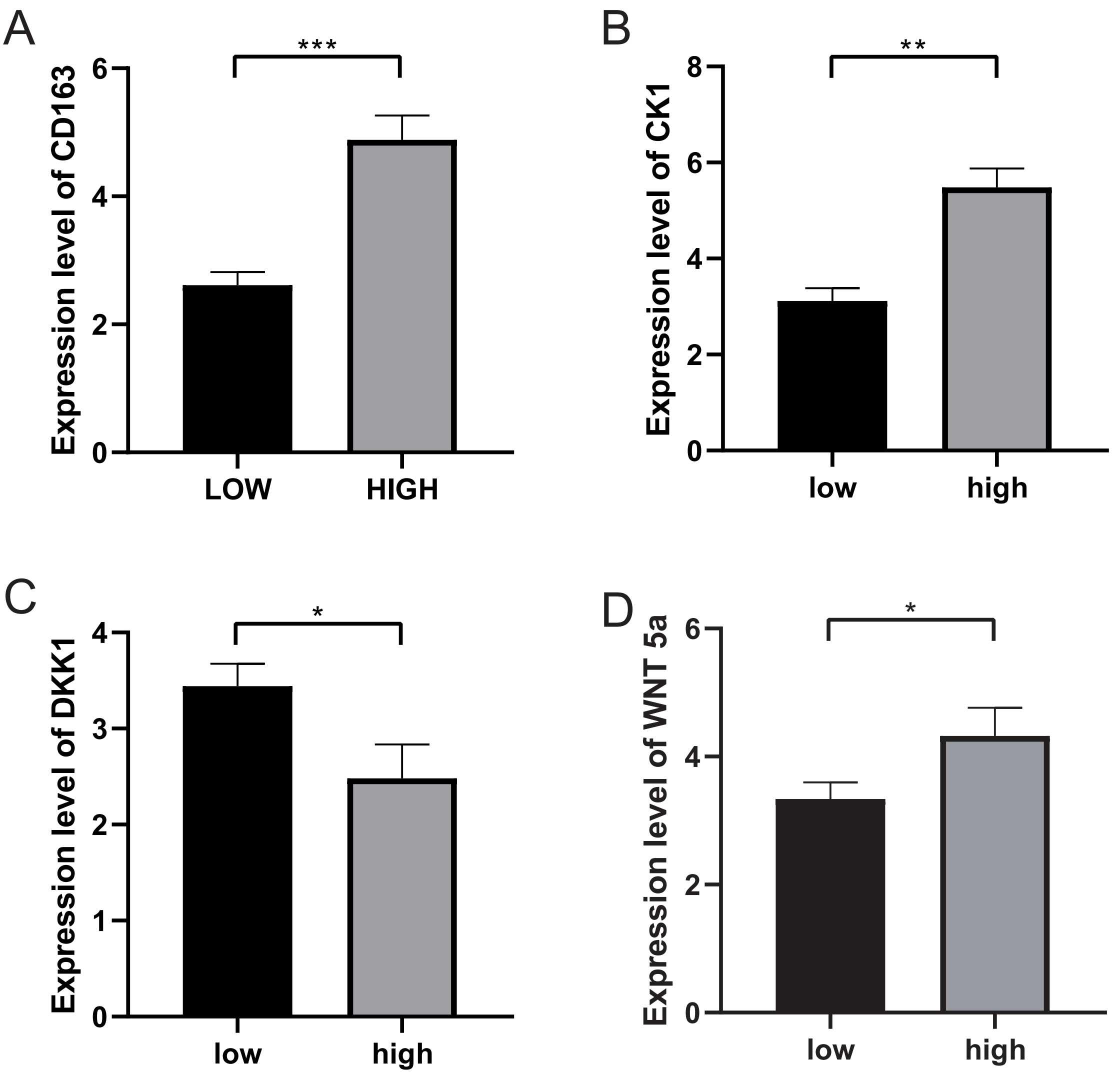

Figure S3. Immunohistochemical data of CD163(A),CK1(B),DKK1(C) and WNT5a(D) in low-and high-grade tumor budding groups.n(LOW)=59,n(HIGH)=25.
