# Supplementary material for: Establish a novel tumor budding-related signature to predict prognosis and guide clinical therapy in colorectal cancer
Source: Sci Rep. 2024 Jan 25;14:2180. doi: 10.1038/s41598-024-52596-1 (PMC10810877; doi:10.1038/s41598-024-52596-1)
Supplement: Supplementary file 4 — Supplementary Table S1. [file 41598_2024_52596_MOESM4_ESM.pdf]

Table S1. The clinicopathological parameters between CRC patients with high- and low-risk group in TCGA cohort.

Characteristics	High-risk n=143	Low-risk n=199	***p***
Gender			0.208
female	62 (43.4%)	100 (50.3%)	
male	81 (56.6%)	99 (49.7%)	
Primary site			0.519
Colon	92 (64.3%)	139 (69.8%)	
Rectosigmoid junction	24 (16.8%)	26 (13.1%)	
Rectum	27 (18.9%)	34 (17.1%)	
pT			0.315
pT1 or pT2	22 (15.4%)	39 (19.6%)	
pT3 or pT4	121 (84.6%)	160 (80.4%)	
pN			0.001
pN0	58 (40.6%)	118 (59.3%)	
pN1 or pN2	85 (59.4%)	81 (40.7%)	
Tumor stage diagnoses			0.002
I/II	57 (39.9%)	113 (56.8%)	
III / IV	86 (60.1%)	86 (43.2%)	
