# Supplementary material for: Establish a novel tumor budding-related signature to predict prognosis and guide clinical therapy in colorectal cancer
Source: Sci Rep. 2024 Jan 25;14:2180. doi: 10.1038/s41598-024-52596-1 (PMC10810877; doi:10.1038/s41598-024-52596-1)
Supplement: Supplementary file 5 — Supplementary Table S2. [file 41598_2024_52596_MOESM5_ESM.pdf]

Table S2. The protein expression in IHC between CRC patients with high- and low-grade tumor budding.

	Tumor Budding		
	low-grade	high-grade	*p*
CK1-low	47	12	0.004
CK1-high	12	13	
DKK1-low	45	23	0.093
DKK1-high	14	2	
WNT5a-low	46	16	0.183
WNT5a-high	13	9	
CD163-low	56	10	<0.001
CD163-high	3	15	
