# Supplementary material for: Establish a novel tumor budding-related signature to predict prognosis and guide clinical therapy in colorectal cancer
Source: Sci Rep. 2024 Jan 25;14:2180. doi: 10.1038/s41598-024-52596-1 (PMC10810877; doi:10.1038/s41598-024-52596-1)
Supplement: Supplementary file 6 — Supplementary Table S3. [file 41598_2024_52596_MOESM6_ESM.pdf]

Table S3.Comparison of M2-like macrophages between high and low tumor budding groups

	Macrophages M2		
	low-group	high-group	*p*
budding low-grade	35	24	0.050
budding high-grade	9	16	
