# Supplementary material for: Establish a novel tumor budding-related signature to predict prognosis and guide clinical therapy in colorectal cancer
Source: Sci Rep. 2024 Jan 25;14:2180. doi: 10.1038/s41598-024-52596-1 (PMC10810877; doi:10.1038/s41598-024-52596-1)
Supplement: Supplementary file 7 — Supplementary Table S4. [file 41598_2024_52596_MOESM7_ESM.pdf]

Supplementary Table S4: Primers used for RT-qPCR.

Genes	Sequence（5'-4')
SAP18	GATCGACCGCGAGAAGACAT
	GAACTCGTCCATTCGGTGGT
MZT1	AGACCATGGACGTTCTGCTT
	GTAGCCTTGCGAAGCTCCTT
POLR1D	CTGGGAACAAAGGAAAGCAATAG
	GAGGACACTTCATCCAACCCAT
MTIF3	CCAGCCCCACTGAGCTTTTC
	TAGAGAAGAATGACAGCTGCAGAA
